# Topology regulatory elements: From shaping genome architecture to gene regulation

**DOI:** 10.1016/j.sbi.2023.102723

**Published:** 2023-11-04

**Authors:** Liang-Fu Chen, Hannah Katherine Long

**Affiliations:** 1 Department of Chemical and Systems Biology, Stanford University School of Medicine, Stanford, CA 94305, USA; 2 MRC Human Genetics Unit, Institute of Genetics and Cancer, University of Edinburgh, Crewe Road, Edinburgh, UK

## Abstract

The importance of 3D genome topology in the control of gene expression is becoming increasingly apparent, while regulatory mechanisms remain incompletely understood. Several recent studies have identified architectural elements that influence developmental gene expression by shaping locus topology. We refer to these elements as topological regulatory elements (TopoREs) to reflect their dual roles in genome organisation and gene expression. Importantly, these elements do not harbour autonomous transcriptional activation capacity, and instead appear to facilitate enhancer-promoter interactions, contributing to robust and precise timing of transcription. We discuss examples of TopoREs from two classes that are either dependent or independent of CTCF binding. Importantly, identification and interpretation of TopoRE function may shed light on multiple aspects of gene regulation, including the relationship between enhancer-promoter proximity and transcription, and enhancer-promoter specificity. Ultimately, understanding TopoRE diversity and function will aid in the interpretation of how human sequence variation can impact transcription and contribute to disease phenotypes.

## Diverse non-coding functional elements regulate gene expression

Regulatory elements within the non-coding portion of the genome play important roles in mediating the timing, location and levels of transcription from target genes. The importance of these elements is highlighted both by Mendelian genetic disorders driven by non-coding mutations that disrupt regulatory elements [1,2] and genome-wide association studies for complex traits which find that most contributing variants are in the non-coding genome [3,4]. One of the most well-studied classes of regulatory elements are enhancers, autonomous cis-regulatory sequences that encode for clusters of transcription factor binding sites that can activate gene expression at a distance in a tissue-specific manner [5–7]. Enhancers can function outside of their native context to drive the expression of a reporter gene, therefore one strategy for identifying enhancers has been to use episomal reporter assays including luciferase assays, *in vivo* transgenic reporters or massively parallel reporter assays (MPRAs) [8,9]. However, it is becoming increasingly apparent that regulatory elements lacking autonomous activity, that would be overlooked in these assays, can also act to facilitate or boost classical enhancers (e.g. [10–13]), or can regulate gene expression by altering 3D genome topology.

In this review, we will focus on an emerging class of architectural elements with a proposed direct function in facilitating enhancer-promoter interactions and gene expression. We refer to these regions as topological regulatory elements (TopoREs), genetically encoded sequences that facilitate regulation of gene expression without having autonomous enhancer activity, by supporting enhancer-promoter communication or otherwise impacting 3D chromatin folding (see Box 1). These TopoREs can be within, close to, or 10s of kilobases away from enhancers or promoters, and their function can span across topologically associated domains (TADs). We will explore in detail several recent examples of TopoREs that shape 3D genome architecture, and impact gene expression, categorised by their dependence on one particular trans-acting factor, CTCF. Finally, we discuss how these elements update our view of the importance of genome structure for gene expression and human disease.

## CTCF-dependent topological regulatory elements facilitate long-range gene regulation

**Figure 1:**
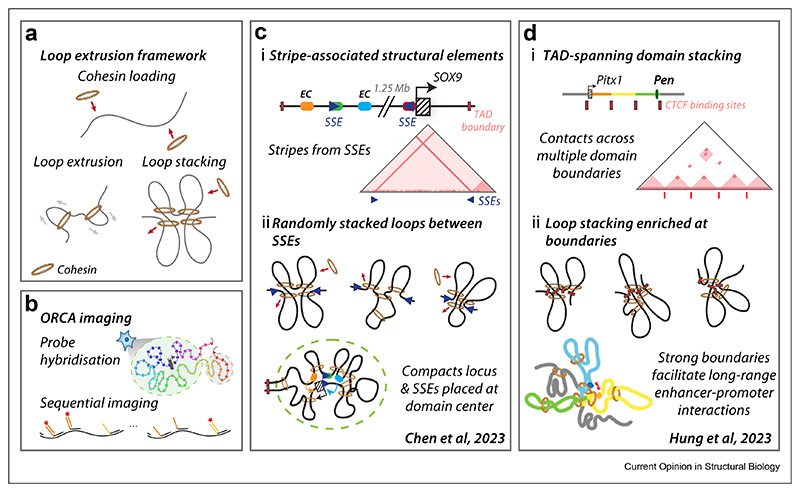
Loop extrusion, CTCF and loop-stacking promote enhancer function across extreme distances. **(a)** Schematic of loading of cohesin onto chromatin followed by loop extrusion. Loop extrusion is halted when cohesin collides with loop extrusion barriers or another cohesin complex. **(b)** Tracing chromatin conformation by optical reconstruction of chromatin architecture (ORCA). A locus of interest is labelled with primary probes, with each probe-set marking desired segments along the locus distinguished with a unique barcode. Each barcode is imaged by sequentially introducing a readout oligo carrying a fluorophore. The 3D structure of the locus then reconstructed after rounds of imaging. **(c)** i) Chen et al. imaged the *SOX9* locus in human cranial neural crest cells and observed stripes at two stripe-associated structural elements (SSEs). ii) These stripes are proposed to form through a multi-loop model whereby loops stack wherever extruding cohesins happen to collide into one another across the domain, anchored at the SSEs. SSEs compact the TAD, draw the *SOX9* promoter into the centre of the 3D domain, and thereby facilitate the promoter interacting with enhancers across the domain. *(d)* i) At the *Pitx1* locus in mouse hindlimbs, Hung et al. observed contacts of the *Pitx1* gene spanning across two TAD boundaries to the distal Pen enhancer. ii) This TAD-spanning interaction is proposed to occur through stacking of TAD boundaries that bring the distal enhancer and promoter that are adjacent to TAD boundaries into close proximity. EC = enhancer cluster.

CCCTC-binding factor (CTCF) is an 11-zinc finger DNA-binding protein that plays a number of important functions including in VDJ recombination and organising 3D chromatin architecture [14,15]. CTCF modulates chromatin organisation together with cohesin, a ring-shaped complex that compacts chromatin through the active process of loop extrusion [16] (Figure 1a). In this process, cohesin is loaded onto chromatin and processively extrudes loops of DNA until stalled through collision with a barrier, such as CTCF bound to DNA in a convergent orientation [17,18]. Through its interactions with cohesin, CTCF has been implicated in a range of topological functions, for example in the formation of topologically associating domains (TADs) and insulated neighbourhoods, which broadly act to constrain regulatory activity within a given locus [19].

CTCF binding within gene regulatory elements has been further implicated in playing a more direct role in facilitating interactions between promoters and enhancers. For example, in mouse Th2 cells, CTCF binding at enhancers conferred an increased tendency for interaction with promoters, and buffered transcriptional noise [20]. Furthermore, in mouse embryonic stem cells (mESCs), CTCF bound at promoters was shown to facilitate enhancer-mediated gene regulation, especially across long distances for genes without many enhancers in close proximity [21]. Mechanistically, CTCF sites adjacent to or within these regulatory elements may facilitate loop extrusion-dependent scanning across the regulatory domain. Transcription factors [22,23] or RNA polymerase II [24] at another distal regulatory element could then stall cohesin to facilitate long-range linking between the enhancer and promoter to promote gene expression. Together, for a subset of genes, CTCF binding at either the promoter or distal enhancer element can provide robustness to gene activation across long-range.

In our recent work, we took an in-depth single-locus approach to explore mechanisms of extreme long-range gene regulation at the *SOX9* locus where craniofacial enhancer clusters (ECs) lie over 1.2 megabases upstream of the *SOX9* gene [25,26]. Using optical reconstruction of chromatin architecture (ORCA) imaging [27] (Figure 1b), and plotting ensemble–average interaction frequencies across the domain, we identified two stripes of domain-spanning interactions emanating from the *SOX9* promoter and EC locus (Figure 1c–i). These elements, which we named “stripe associated structural elements” (SSEs), were dependent on CTCF-binding sites for both topological function and for maintaining normal expression levels of *SOX9* [25]. The single chromatin fibre nature of the ORCA imaging enabled us to observe hugely dynamic and variable locus topologies for cell states across our *in vitro* differentiation time course, from human embryonic stem cells to cranial neural crest cells (Figure 1c-ii). We identified that the differences between averaged *SOX9* domain structures for these cell states arose from alterations of sampled frequencies in domain topologies rather than a shift between two static preferred structures. The ORCA single-fibre topologies further allowed us to determine that SSEs promote the positioning of the *SOX9* gene in the geometric centre of the domain. We propose that this locus topology facilitates interaction of the promoter with the entire TAD. While a number of mechanisms may be at play to drive SSE function, we explored the role of loop extrusion through polymer simulations and determined that a multi-loop structure was consistent with the chromatin fibre topologies we observed, where multiple extruded loops stack across the domain, bridging the long distance between the distal enhancers and the *SOX9* gene (Figure 1c). Ultimately, we propose that this conformation facilitates gene regulation by promoting sampling of the regulatory domain by the *SOX9* promoter. Interestingly, similar stripe-like features were previously noted from Capture Hi-C in E12.5 mouse limb buds [28]. Future singular deletion of the orthologous SSE1.35 element in mouse development will help to reveal cell-type specificity and evolutionary conservation of SSE1.35 function.

Stacking of loops as a mechanism for extreme long-range regulation has been further extended recently to span across multiple TADs or contact domains [29]. TADs or contact domains have been broadly defined as domains of higher intra-region contact with reduced inter-region contact, seen as a triangle on the diagonal in HiC heatmaps [30,31]. Boundaries between these domains have been considered to both facilitate intra-domain interactions and insulate genes from the regulatory influence of enhancers from adjacent domains. Indeed, patient mutations perturbing these boundary elements are associated with disease through the resultant misregulation of target genes [2,32,33]. Controverting this paradigm, there are examples of enhancer action spanning across domain boundaries. For example, a distal super-enhancer at the *Hoxa* locus is important for ear development that functions across a TAD boundary [34]. Additionally, at the *Pitx1* locus, the Pen enhancer regulates *Pitx1* gene expression across long distance in mouse hindlimb development, traversing three self-interacting contact domains [35]. In this second example, ORCA imaging revealed multi-way interactions between boundary elements in a single chromatin fibre. This boundary hub is thought to bring the boundary-proximal enhancer and promoter into close proximity. Therefore, while the intervening domain boundaries between *Pitx1* and the Pen enhancer insulate contacts between the self-interacting domains, they are also proposed to facilitate long-range enhancer function through domain boundary-stacking (Figure 1d). Given that they facilitate enhancer-promoter interaction through modulation of 3D genome folding, we propose that in this case the *Pitx1* locus domain boundaries are behaving as TopoREs. While many boundary elements may not meet our criteria as a TopoRE, a prediction of this model is that enhancers and promoters located near domain boundaries are more likely to be subject to this type of regulation. These observations therefore lay the ground for other extremely distal enhancer-promoter pairs to be identified, and provide a framework for understanding other examples of domain-spanning enhancer action.

CTCF plays diverse roles in the control of 3D chromosome topology and gene regulation; however, it remains poorly understood how different CTCF sites act in a distinct manner to shape local chromosome topology. For example, at the *SOX9* locus, there are many more sites bound by CTCF than those required for SSE function [25]. Possible contributing factors influencing CTCF topological function at distinct sites include co-binding of other trans-regulatory factors [36,37], location of extruder loading, proximity of regulatory elements to the CTCF bound region, and the affinity of CTCF binding to the element itself. It is conceivable that these properties could then be regulated across diverse tissue types and developmental stages to change the nature of a topological regulatory element, for example from an insulator to a structural element facilitating enhancer-promoter interactions. It remains to be seen therefore whether CTCF sites involved in mediating enhancer-promoter interactions play a pleiotropic regulatory role across all tissues where active gene regulation is occurring, or whether TopoRE elements exhibit cell-type specificity as is seen for enhancers.

## Interplay between boundary elements and CTCF-independent topological regulatory elements provide specificity and precise timing for developmental gene expression

**Figure 2:**
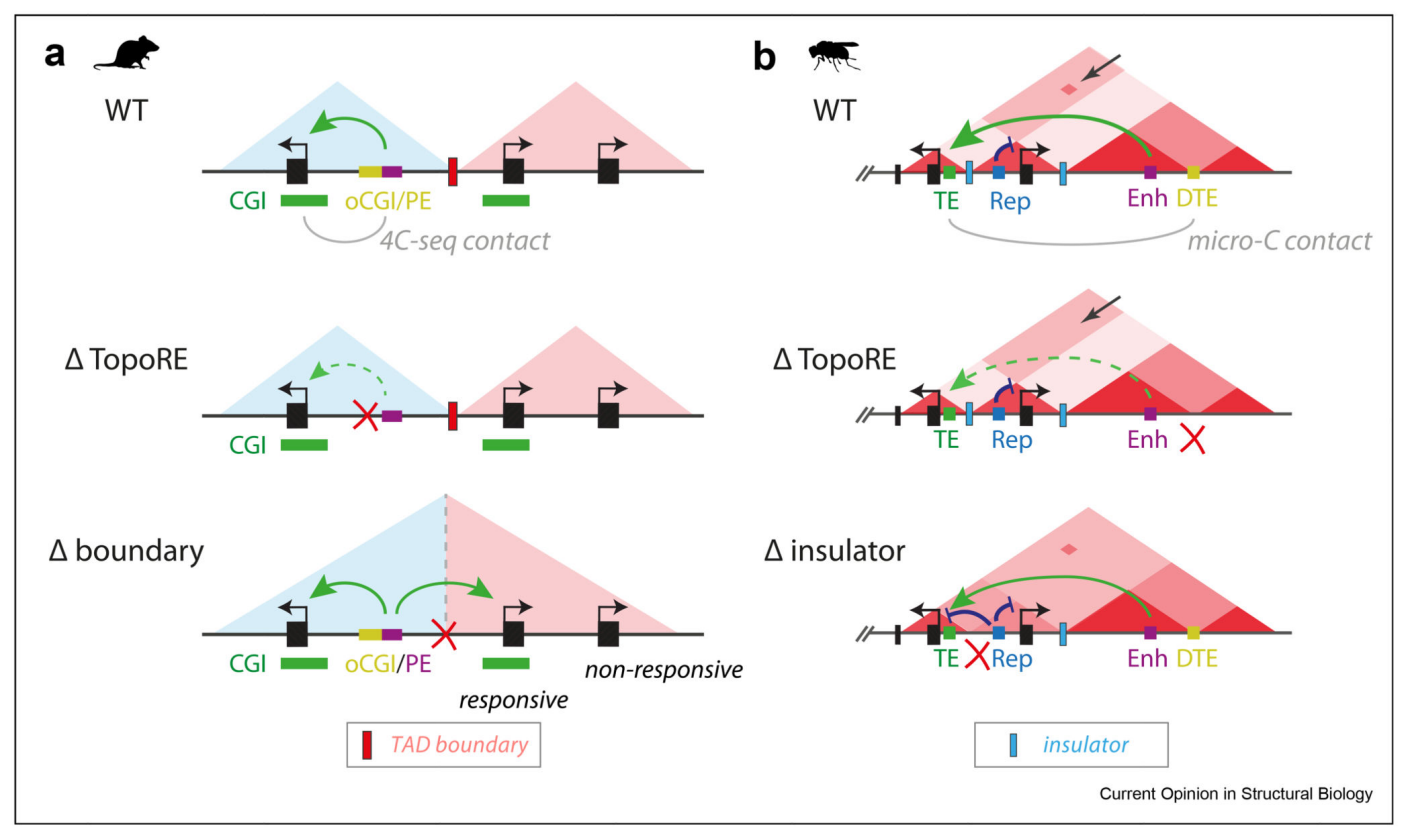
Interplay between topological regulatory elements and boundary elements drives gene expression specificity. **(a)** In mouse embryonic stem cells, orphan CpG islands (oCGIs) help to bridge poised enhancers to target promoters embedded within CGIs, as detected by 4C-seq. This is mediated by polycomb in mESC and proposed to be bridged by transcription factors upon transcriptional initiation after differentiation to anterior neural progenitors (AntNPCs). Loss of oCGI elements cause a reduction in this interaction, and a reduction of target gene expression during differentiation to anterior neural progenitors (AntNPCs). While loss of TopoRE (oCGI) leads to a reduction in gene expression, loss of the TAD boundary can drive misexpression of another gene embedded within a CGI in the adjacent TAD due to interaction compatibility with the nearby oCGI/PE. **(b)** During *Drosophila* development, a tethering element (TE) at the *Scr* gene interacts with a distal tethering element (DTE) near an enhancer, as detected by Micro-C, bypassing an intervening self-interacting domain. Ablation of the DTE leads to a delayed developmental expression of *Scr*, while ablation of an intervening insulator element enables a regulatory element (Rep, AE1) to interact with the *Scr* gene also leading to transcriptional downregulation of *Scr*.

While many enhancer-promoter interactions have been shown to be mediated by CTCF and cohesin, there are several studies reporting that a distinct group of TopoREs can shape genome structure and regulate gene expression in a CTCF-independent manner. Indeed, the majority of genes are able to recruit enhancers and initiate transcription normally in the face of acute degradation of CTCF [21,38–41]. In mESCs, CpG islands (CGIs) have been shown to promote long-range communication between promoters with large CGIs and poised enhancers (PEs) associated with an orphan CGI (oCGI) [42]. In total, around 60–80 % of PEs in mouse ESCs are located within 3 kilobases (kb) of an oCGI and deleting oCGIs at PEs reduces the expression of their target genes. However, these oCGIs do not increase the transcriptional activity of PEs. Instead, they facilitate PE interactions with target genes, and only promoters with large CGI clusters show a transcriptional responsiveness to PEs. These CGI-mediated interactions can be blocked by TAD boundaries and thus it was proposed that the combination of CGI-mediated long-range communication and the insulation from TAD boundaries provides specificity in the induction of certain genes during development [42] (Figure 2a).

A similar interplay between boundary elements and TopoREs has also been shown to shape the specificity and timing of developmental gene expression during *Drosophila* development. Leveraging high-resolution Micro-C data, Batut et al. identified two distinct classes of architectural elements that shape genome structure and regulate gene expression during a critical 60 minutes of development prior to gastrulation [43]. Insulators act to prevent spurious interactions, while distal tethering elements (DTEs) foster appropriate enhancer-promoter interactions. One-third of all focal contacts detected by Micro-C connect promoters of protein-coding genes to DTEs, typically spanning tens of kilo-bases. Tethering elements identified at the *Scr-Antp* region overlapped with regions previously identified as facilitating enhancer-promoter selectivity for the *Scr* gene [44,45]. Overall, DTEs were observed at many critical developmental loci reflecting a potentially broad mechanism for mediating enhancer-promoter interactions important for transcriptional timing. DTE elements display no autonomous enhancer activity in the early embryo. However, using live cell imaging of transcription, the authors demonstrated that DTEs foster fast activation of transcriptional kinetics required for appropriate developmental progression, while boundaries prevent interference of cis-regulatory elements between neighbouring TADs. Therefore, DTEs meet our criteria as topological regulatory elements and the interplay between boundaries and DTEs is proposed to confer the specificity and timing of developmental gene transcription in the developing *Drosophila* embryo (Figure 2b).

**Figure 3:**
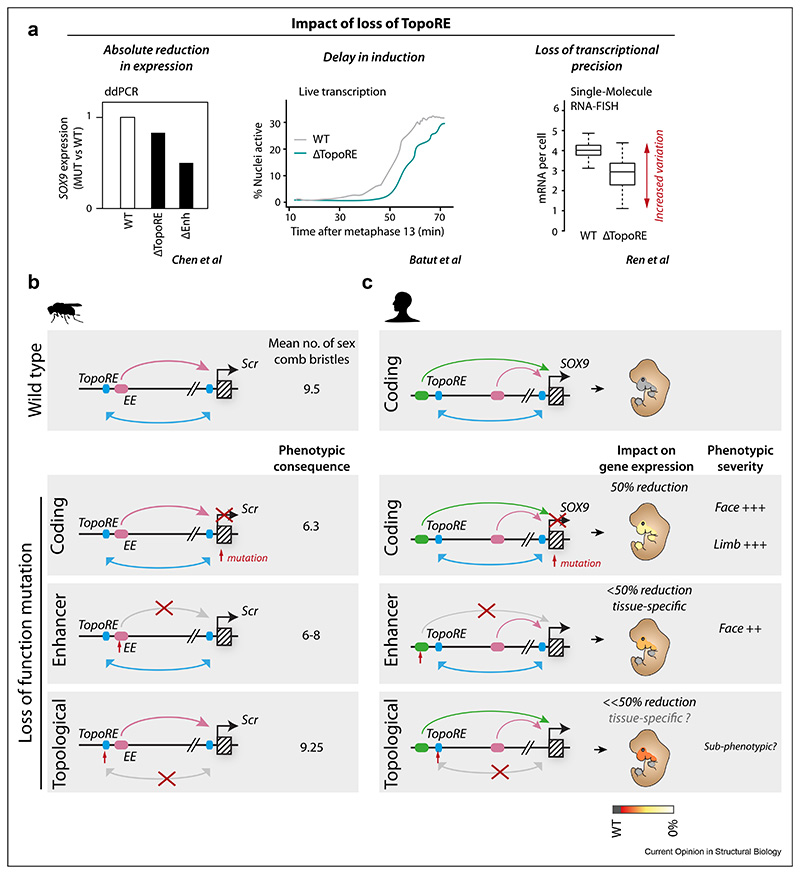
Transcriptional and phenotypic consequences of topological regulatory element perturbation. **(a)** Perturbation of a TopoRE can impact absolute expression levels (left, example of *SOX9* locus Chen et al.), transcriptional timing (middle, Batut et al. Pachano et al.) or can lead to loss of transcriptional precision (right, Ren et al.). **(b)** In wildtype *Drosophila* embryos, developmental expression of *Scr* is required for a normal number of sex comb bristles (a mean of 9.5), and heterozygous loss of *Scr* reduces this to an average of 6.3. Heterozygous loss of the distal Scr enhancer element (EE) reduces *Scr* expression, leading to fewer sex comb bristles (6–8 on average). Perturbation of a distal tethering element (DTE) adjacent to the EE enhancer leads to a delay in Scr induction (see A) and a subtle reduction in sex comb number. **(c)** At the *SOX9* locus, stripe-associated structural elements (SSEs) facilitate *SOX9* expression in cranial neural crest cells. Heterozygous loss of *SOX9* function impacts all *SOX9* expressing tissues (simplified here to show face and limb expression) leading to severe phenotypes in both tissues. Loss of craniofacial distal enhancer elements (e.g. EC1.45) reduces *SOX9* expression only in the face, leading to phenotypes in PRS patients restricted to the lower jaw. Mutation of the SSE elements have a milder impact on *SOX9* expression in CNCCs, and it is predicted this may have sub-phenotypic consequences during development but may sensitise embryonic development to other environmental or genetic perturbations. Whether the SSE elements are tissue-specific in their function remains to be determined.

Orthologous to CTCF-dependent TopoREs, diverse transcription factors/cofactors bind to CTCF-independent TopoREs and can regulate their functions in different cell types or developmental stages. While the dynamics of locus topology and constant re-establishment of TAD structure are becoming apparent [25,46,47], Pachano et al. observed that PEs/oCGIs are already in close proximity on average to their target promoter/CGI before gene activation. These interactions are dependent on polycomb complexes in mESCs [48] and are proposed to be maintained by transcription factors and co-factors once the PEs become active in anterior neural progenitor cells [42]. Similarly, DTEs in Drosophila are bound by pioneer factors such as Trithorax-like (Trl), grainyhead (grh), and zelda (zld) [43], which appear to mediate enhancer-promoter interactions prior to gene activation. Indeed, zld has been shown to mediate cis-regulatory chromatin interactions that arise before the formation of TADs and gene activation during early *Drosophila* development [49]. Importantly, this binding of CTCF-independent TopoREs by different transcription factors/cofactors appears to shape genome structure for subsequent gene expression during development. These TopoREs therefore promote the frequent sampling of permissive regulatory topologies whereby enhancers are already in proximity to their target genes prior to gene activation to ensure precise timing of developmental gene expression once the enhancer is turned on (Figure 3a). Of note, this is reminiscent of the observed proximity seen to link the *Shh* gene to the distal ZRS limb enhancer in non-expressing cell types [50–52]. Together, TopoREs provide an extra layer for gene regulation, with the interplay between TopoREs and boundary/insulator elements providing specificity and precision of timing for developmental gene expression.

## The importance of 3D chromatin organisation for gene expression and human disease

Above, we have described a breadth of topological regulatory elements that influence developmental and homeostatic gene expression through an influence on 3D genome folding and enhancer-promoter communication. At the *SOX9* and *Shh* regulatory loci, loss of CTCF-binding sites leads to a general increase in pairwise distances across the domain [25,52]. Therefore, a key role of TopoREs may be also to compact a regulatory locus to promote the frequency of enhancer-promoter interactions. This role of TopoREs may be most relevant for the activity of distal enhancers, as recent studies have revealed a differing requirement for cohesin for activation from proximal versus distal enhancers [53,54]. This suggests a distinct requirement for enhancer-promoter tethering or locus compaction for enhancer function across different genomic distances. In this context, TopoREs may play a greater role in facilitating gene regulation for more distal regulatory interactions, while enhancer-promoter proximity is less of a limiting feature for proximal regulatory elements. Of note, a number of mechanisms have been proposed for enhancer-promoter communication which are independent of CTCF or TopoRE function, including transcription factor-mediated interactions or diffusion of modified factors from an active enhancer to a promoter [5,23,55,56].

An understanding of the role of TopoREs in development is of great importance in the context of a full appreciation of the impact of non-coding mutations on human disease. Increasing evidence points to a critical role of 3D chromatin organisation during organismal development and cell differentiation, and gross deregulation of chromatin topology and TAD architecture is associated with the development of human diseases [33,57]. In the context of TAD boundary perturbations (e.g. due to a structural variant), a TopoRE could drive a gain-of-function pathological phenotype by forming novel contacts between enhancers and a non-target gene promoter (e.g. Figure 2a, lower) [42]. Furthermore, it is likely that disruption of TopoRE function could also deregulate gene regulation to such an extent as to cause developmental defects and disease. For example, the importance of CTCF for facilitating enhancer-promoter interactions is underlined by the discovery that patients with acheiropodia harbour mutations ablating a cluster of CTCF sites upstream of the *Shh* limb enhancer, ZRS, which facilitate interaction with the *Shh* gene [58]. In addition, deletion of tethering elements at the *Scr* locus in Drosophila caused a delay of precisely timed developmental gene expression. While the levels of transcription ultimately catch up, this delay impacts sex comb development proportional to the degree of transcriptional impact [43,59–61] (Figure 3a and b). At the *SOX9* locus, ablation of extreme long-range enhancers has a tissue-specific effect on lower jaw development likely due to a combination of tissue-specific dosage sensitivity to *SOX9* perturbation and spatially restricted domains of enhancer activity. While no patients have yet been identified with SSE perturbation alone, ablation of either of the *SOX9* locus structural elements perturbs expression levels in CNCC cell culture models. Loss of SSE function could therefore be sub-phenotypic but sensitise facial development to other genetic or environmental perturbations (Figure 3c). As discussed above, it remains to be seen whether TopoRE loss would have pleiotropic effects on disease or have tissue-specific functions. Future work will be required to determine the topology and SSE status of other *SOX9* expressing cell types.

Due to the lack of autonomous regulatory activity, and in many cases a lack of uniquely bound trans-acting factors that distinguish them from non-regulatory elements, there are currently limited ways to identify TopoREs genome-wide. Reporter assays are dependent on autonomous regulatory capacity, and without a unique molecular signature, ChIP-seq-based methods cannot identify these elements in a high throughput manner. However, as our understanding and discovery of these elements increases, greater in-depth exploration of the genomic and trans-acting determinants of chromatin looping and topological regulatory element activity will further illuminate TopoRE function. As an example, the *Sox2* locus has been intensively studied, and highlights how focussing on a single locus can uncover fundamental features of cis-regulatory landscapes which can then be explored genome-wide. Both mutational screening and synthetic engineering of the *Sox2* locus in mESC have started to uncover the grammar of CTCF-binding sites for CTCF function, as well as other factors that modulate chromatin architecture [12,39,62,63]. These single-locus studies together with CRISPR-based genome editing [64,65] and high-throughput screens for co-factors of CTCF [36] will greatly increase our understanding of TopoREs more broadly. Another way to identify architectural elements in the genome is *in silico* discovery of DNA sequence features that mediate distal interactions by deep learning (DL) models coupled with genome-wide 3C-based sequencing data. Many recent studies have been able to apply DL models to train sequence-based predictors of chromatin looping and to identify specific sequence features that may facilitate physical contacts between distal genomic regions (see reviews from Refs. [66,67]). Extending this analysis to identify features unique to TopoREs, combined with experimental validation, will be a powerful tool to study the DNA sequence grammar underlying TopoREs.

In concert with additional features of genome structure such as domain boundaries and insulators, TopoREs confer robustness and specificity in gene transcription. Ultimately, an improved understanding of how TopoREs are regulated during development will shed light on how alteration of these elements can impact gene expression and contribute to disease phenotypes.

## Data Availability

No data was used for the research described in the article.

## Box 1 Proposed definition and criteria for a topological regulatory element (TopoRE)

We suggest the following features for defining a topological regulatory (TopoRE) element:

- a region of DNA that lacks autonomous enhancer activity (by reporter assay in a relevant cell type);.
- genetic ablation leads to an alteration of 3D genome topology (e.g. enhancer-promoter distance);.
- genetic ablation impacts target gene expression either in the current cell-state or during developmental progression..
